# Teaching the Turkish language to foreigners at higher education level in Northern Cyprus: An evaluation based on self-perceived dominant intelligence types, twenty-first-century skills and learning technologies

**DOI:** 10.3389/fpsyg.2023.1120701

**Published:** 2023-02-27

**Authors:** Esra Karakaş Kurt, Ahmet Güneyli

**Affiliations:** ^1^Department of Turkish and Social Sciences Education, Education Faculty, Cyprus International University, Nicosia, Cyprus; ^2^Department of Turkish and Social Sciences Education, Education Faculty, European University of Lefke, Lefke, Cyprus

**Keywords:** teaching Turkish as a foreign language, higher-education students, twenty-first-century skills, learning technologies, perceived dominant intelligence types

## Abstract

**Introduction:**

There are many foreign students in higher education in Northern Cyprus. Both the academic and life skills of these students depend on attaching the necessary importance to their Turkish language teaching. The goal of this study is to examine how university students employ learning technology, twenty-first-century abilities, and perceived categories of intelligence in the process of learning a foreign language.

**Methods:**

In line with the quantitative research design, this study utilized a descriptive approach. Purposeful and convenience sampling methods were used to create the study sample. As a result, the institution in Northern Cyprus with the largest international student body was chosen. At this university, one of the authors of this study has been employed, and Turkish is the language of teaching. The study sample consisted of 431 university students who took Turkish as a foreign language in the 2021–2022 academic year at the selected university.

**Results:**

The results of the study revealed a weak yet statistically significant correlation between twenty-first-century skills and usage of foreign language-learning technologies. Additionally, students' twenty-first-century skill scores differed significantly, whereas their foreign language-learning technology scale scores did not match their self-perceived intelligence types.

**Conclusion:**

The research's findings indicate that students in higher education possess twenty-first-century skills. Based on this finding, it is possible to engage students in the courses and accomplish effective foreign language acquisition if foreign language education is carried out in accordance with modern methodologies and based on twenty-first-century abilities. It has been revealed in this study that it is important to include social learning rather than individual and competitive learning in foreign language education classes.

## 1. Introduction

Considering that the world is digitalized and globalized more with each passing day, it is self-evident that the number of people who speak a foreign language will increase progressively all over the world as it is in EU countries. It has been stated in the Organization for Economic Cooperation and Development (OECD) Programme for International Student Assessment (PISA) 2018 Report, which is based on data obtained from 75 countries, that more than 95% of students either speak more than one language or are learning at least one foreign language (OECD and PISA, 2018). These data indicate that we need to focus on teaching and learning foreign languages even more. No matter how foreign language teaching or learning is conducted, taking into account the current state of technology, enriched, interactive digital resources and the digital platforms where these materials are presented rank among the most crucial tools in the process. Today, there are rich contents and various tools (z-books, digital games, speech bots, web 2.0 tools, etc.) that can be used by both foreign language teachers and learners for the improvement of reading, listening, writing, and speaking skills. In short, as in all other areas of life, all learning-teaching activities at school are affected by technology and media-oriented lives. In light of this, it is evident that modern individuals require functional skills such as media literacy, information literacy, and computer and information technology (Partnership for 21st Century Learning, 2021).

The pandemic caused by the coronavirus disease (COVID-19) has brought about a compulsory transformation in education. In this context, it has been observed that learning technologies and virtual environments were particularly effective on the learning skills of students who were confined to their homes (Adedoyin and Soykan, 2020). While technology-supported teaching skills or competencies of teachers were of more importance before the pandemic, the issue of how students can learn more effectively by using technology has gained more importance with the pandemic (Daniel, 2020).

Recent studies on foreign language education demonstrated that there remains a strong and growing demand for employees with high language and cultural competencies in both the private and public sectors, notably in healthcare, social services, translation and interpretation services, travel, and tourism sectors (Damari et al., 2018; Looney and Lusin, 2018). However, studies have also shown that the foreign language-learning process is not efficient enough to meet this demand of the business sector (Stein-Smith, 2016; Quicios, 2018). Studies conducted on students revealed that traditional approaches tend to dominate the foreign language-learning process. For example, although there is a tendency toward adopting learner-centered approaches in education in general, traditional, and conceptual approaches continue to be used frequently in language education (Kim, 2019).

It is especially important for foreigners who come to study in Northern Cyprus, which has a multicultural and multilingual structure, to learn Turkish language not only for their academic life but also for them to continue their daily lives without any problems. In fact, the number of international students studying in the universities of Northern Cyprus, which is located in the northeast of an island in the Mediterranean Sea, is even more than the number of domestic students. Statistical data announced by the Ministry of National Education of Northern Cyprus for the 2020–2021 academic year indicated that a total of 103,108 university students have been studying in Northern Cyprus and only 13% (13,427) of these students were Cypriots. Students coming from Turkey constitute the largest group of international students, followed by students coming from the African continent (Ministry of National Education Culture of North Cyprus, 2021). In a study by Osmanli (2018), it was noted that international students make up almost half of the population in some of the cities in Northern Cyprus. For example, in 2021, the population of local residents of Nicosia, the capital of Northern Cyprus, was 61,376, and the number of international students was 41,416. Thus, international students accounted for ~40% of the city's population. Therefore, as also stated in several studies available in the literature (Gülmez, 2018; Yücel, 2018), the universities in Northern Cyprus have both a multilingual and multicultural structure. The aim of this study is to assess how well university students in Northern Cyprus who have a sizable international student population are learning Turkish as a foreign language.

“Self-perceived intelligence type” is the first factor taken into account when conducting research on this subject. Breakspear (2013, p. 692) explains the new definition of the intelligence in his article as “*Intelligence is a corporate capability to forecast change in time to do something about it. The capability involves foresight and insight, and is intended to identify impending change which may be positive, representing opportunity, or negative, representing threat.”* In this study, self-perceived intelligence areas are the types of intelligence defined by Gardner's Multiple Intelligence Theory. Gardner proposed seven different intelligence dimensions in his book “Frames of Mind” published in 1983. Later, in his work titled “Intelligence Reframed” published in 1999, he added the new intelligence dimension and created eight different intelligence dimensions. These are; Verbal-linguistic, Logical-mathematical, Visual-spatial, Musical-rhythmic, Bodily-kinesthetic, Interpersonal, Intrapersonal, and Naturalistic. Karadag and Baştug (2018) reveal that despite the increasing rate of mental assessment, intelligence is still not evaluated more intelligently in Turkey. One of the reasons for this is the problems related to the training and competence of psychologists who apply the intelligence tests. It is observed that families encourage their children to take intelligence tests not for a clinical purpose but because of their personal curiosity. It is also noted that the principle of being beneficial or not harming is not implemented much in Turkey. For example, based on the intelligence test results, it is decided whether a student will receive inclusive education or not. A wrong decision can lead to an education that is not suitable for the level of the student. Furthermore, it is revealed that ethical principles such as responsibility, respect for human rights and non-discrimination are sometimes not taken into consideration. As an alternative to these problems, as Salman et al. (2017) stated, psychologists and educators suggest that Gardner's theory of intelligence can be used in education. The experts state that the theory of multiple intelligences is objective, the level of the students is not graded and the students are not labeled as sufficient or insufficient. Valuing intelligence types other than mathematical and verbal intelligence types is also considered as an important feature of multiple intelligences. In multiple intelligence theory, it is stated that the tests do not necessarily have to be administered by experts and this situation creates an ease of application for teachers, parents and psychological counselors. It is highlighted that it is an important acquisition for a person who makes a self-evaluation in the fields of multiple intelligences to be aware of their own abilities and skills.

Gardner's point of view on the concept of intelligence, which has been highly criticized and controversial in the scientific world, has led to mobility in the field of education and training. In 1983, Gardner argued in his book Frames of Mind that there is no single intelligence measured by the well-known IQ test, otherwise termed “g.” Gardner added that IQ is not exclusively assessed by standardized testing. The many intelligences theory can be used to help someone choose the best learning method for them. According to some psychologists, the multiple intelligence theory is not acknowledged as a valid theory and is not considered to be a universal instrument for explaining human cognitive skills (Waterhouse, 2010; Sternberg, 2015). However, it is very important for a person to be aware of their own abilities and to be able to know themselves. For this reason, Gardner's theory has been especially selected and it aims to contribute to the literature with the findings revealed in the research.

The Theory of Multiple Intelligences asserts that intelligence is multifaceted and aims to improve the existing abilities and potentials of individuals. Traditional educational approaches which are based only on verbal-linguistic and logical-mathematical intelligence fields, have been eliminated increasing the diversity in education. According to the Theory of Multiple Intelligences, students who are successful in intelligence areas other than verbal-linguistic and logical-mathematical intelligence areas can also be described as successful or intelligent. Seven different intelligence types were defined in Gardner's multiple intelligence theory (Gardner, 1983). Gardner later defined another intelligence type, making a total of eight: verbal–linguistic, logical–mathematical, visual–spatial, musical–rhythmic, bodily–kinesthetic, interpersonal, intrapersonal, and naturalistic (Gardner, 1999). According to Gardner, cognitive abilities are independent of each other; therefore, there can be different intelligence types depending on the cognitive domains. Studies on intelligence types have also been undertaken in the field of education. According to the multiple intelligence theory, students have different intelligence types and their learning is affected by the dominant intelligence type they have (Zebari et al., 2018; Lei et al., 2021; Gandasari et al., 2022; Wreede, 2022). The theory of multiple intelligences asserts that intelligence is multifaceted. Traditional educational approaches which are based only on verbal–linguistic and logical–mathematical intelligence fields, have been eliminated, increasing the diversity in education. According to the theory of multiple intelligences, students who are successful in intelligence types other than verbal–linguistic and logical–mathematical intelligence types can also be described as successful or intelligent (Keskin, 2019).

Gardner's theory has also been discussed in the literature in the context of teaching Turkish as a foreign language. In one of these studies, Keskin (2019) reviewed the course material used in teaching Turkish as a foreign language (Yedi Iklim Turkish Teaching Set) in terms of the multiple intelligence theory and determined that the Yedi Iklim Turkish Teaching Set utilized the verbal–linguistic intelligence area the most; however, it did not equally address the remaining seven intelligence types. In another study, Çökmez (2017) determined that Turkish language teaching materials addressed verbal–linguistic and logical–mathematical intelligence areas at a rate of 59.1 and 35.2%, respectively. Creating different activities to develop intelligence types that are little used or not used at all has been suggested. As stated in the literature, it is emphasized that there are some problems due to the use of multiple intelligence theory in teaching Turkish as a foreign language. For this reason, Gardner's theory has been specially selected, and this study aims to contribute to the literature with the findings revealed in the research.

## 2. Conceptual framework

Intelligence in foreign language education is associated with the cognitive dimension when evaluated theoretically. Güneş (2011) suggests that, besides the cognitive dimension, behavioral, and constructivist theories are important in foreign language education. Based on the behavioral language education theory, language-learning technologies have been discussed and researched in the literature. In this study, language learning technologies were defined using Hayta's (2014) study. Accordingly, language learning technologies are computers, internet, media, and mobiles technologies. The tools used in these learning technologies are; movies, short videos, online dictionaries, songs, grammer/exercise websites, podcasts, audio books, short stories and novels on computers, journals and newspaper on the internet, social communication networks (Skype, facebook, twitter, whatsApp, video calling etc.), translation facilities on the internet (Google translations). On the basis of the constructivist language education theory, it can be seen that one of the current issues, that of twenty-first-century skills, has drawn attention. In this study, twenty-first-century skills are defined on the basis of Eker's (2020) study. Ac-cording to this; Communication and Collaboration (Communicate Clearly, Collaborate with Others, Think Interdependently), Creativity and Innovation (Think Creatively, Work Creatively with Others, Apply Past Knowledge to New Situations), Critical Thinking and Problem Solving (Think Critically, Make Judgments and Decisions, Ask Questions, Solve Problems), Reflection and Awareness, (Metacognition/Thinking about Our Thinking, Reflect and Synthesize). Thus, language-learning technologies and twenty-first-century skills are investigated in addition to the intelligence type variable in this research. These two variables are important in teaching Turkish as a foreign language, as pointed out in the literature.

Kalemkuş and Özek (2021) conducted content analysis on 115 studies on twenty-first-century skills carried out between 2000 and 2020 and found that the Turkish language curriculum, Turkish teachers, Turkish teacher candidates and Turkish-language textbooks were evaluated based on twenty-first-century skills, but teaching Turkish as a foreign language was not. Dündar and Polat (2021) investigated teaching Turkish as a foreign language within the scope of twenty-first-century skills of the curriculum, including the acquisitions of listening, spoken interaction, spoken production, reading, and writing skills from A1 to C1 levels, and concluded that twenty-first-century skills, which are key for students developing their competencies, were not sufficiently included in the curriculum. The other issue is about learning technologies in foreign language teaching. Since the 2000s, the number of scientific studies on teaching Turkish as a foreign language has increased. However, the studies that address the technological aspect of the matter are still not of the desired quality and quantity (Güntaş et al., 2021). It is important to follow current and technological developments in education in order to increase the quality of language teaching and the active participation of students.

### 2.1. Learning technologies in foreign language education

Foreign language learning is the most suitable field of education for use of information technologies (Ahmadi, 2018). Information technologies aid students in a variety of ways. First, information technologies allow a smooth transition from the traditional model of teacher-centered learning to learner-centered learning. In this way, the individual differences between learners can be addressed and their motivation can be increased as a result.

Using a variety of resources, such as short films, online dictionaries, songs, websites with grammar exercises, podcasts, audiobooks, short stories and novels, journals and newspapers, and social media platforms, it is possible to teach and learn foreign languages successfully (Hayta, 2014). Interactive digital materials (z-books, digital games, speech bots, web 2.0 tools, etc.) and the digital platforms where these materials are presented offer enriched solutions to improve reading, listening, writing, and speaking skills, regardless of the method used for teaching/learning a foreign language. In short, as in all other areas, teaching/learning a foreign language is not outside the scope of technology and media.

Parallel to this, there have been further studies on the use of technology in teaching Turkish as a foreign language as well as in global foreign language education. In this context, integration of technology with language learning–teaching (Birinci, 2020; Repetto et al., 2021; Van Lieshout and Cardoso, 2022), the use of web 2.0 tools, social media, blogs and extracurricular learning environments in language teaching (Bozavli, 2017; Taylan, 2018; Ustabulut and Keskin, 2020; Inal and Arslanbaş, 2021; Sarigül, 2021), digital stories (Akdag and Altinay, 2021; Çokyaman and Çelebi, 2021; Kazazoglu and Bilir, 2021), e-portfolios (Erice and Ertaş, 2011), virtual classrooms (Parmaxi, 2020), and robot teachers (Edwards and Cheok, 2018) have been addressed in the literature. Nevertheless, the results of these studies on the competencies of both teachers and students regarding the use of technology in the language learning–teaching process are contradictory. The discrepancies between these studies may be attributed to the differences between the characteristics of the respective samples since it is known that some individuals easily adapt to the use of technology in the language-learning process, while others show resistance. Indeed, investigating the reasons for these differences between individuals in adapting to the use of technology in the language-learning process and raising awareness about the use of learning technologies in foreign language education were the primary motivational factors for this study.

Many researchers in the field of language education support an open transition to technology-enhanced, student-centered instruction that improves language proficiency (Amini and Amini, 2017; Hong et al., 2017). In addition, these researchers promote the use of a holistic approach in language education that combines language, literature, and culture (Mohr and Welker, 2017; Morska et al., 2018). It is very important for students to actively participate in foreign language classes regardless of the grade level. However, challenging curricular content, contextually inappropriate learning tasks and teaching approaches that fail to involve students as active participants in their learning are reasons why students' active participation cannot be achieved at the levels desired (Philp and Duchesne, 2016; Park and Hiver, 2017). Therefore, language education in general and foreign language education in particular should not focus solely on specific contents, themes and concepts. Rather, language education should prepare students for rapidly changing economic, political, and social conditions and develop their twenty-first-century skills in the ever-changing realities of a globalized society (Moeller and Abbott, 2018; Quicios, 2018). Yeni's (2018) found that twenty-first-century skills increased the educational technology and material development competencies of foreign language teachers.

### 2.2. Twenty-first-century skills

The classification of twenty-first-century skills was made within the framework of the Partnership for twenty-first-century skills (P21). Accordingly, skills have been placed in several categories. The first is “Life and Career Skills,” which includes flexibility, communication and cooperation skills. These skills focus on critical thinking and adaptability, entrepreneurship and self-management, social and intercultural skills, productivity and accountability, leader-ship, and responsibility. The second category is “Learning and Innovation Skills,” which focuses on several dimensions, i.e., critical thinking, communication, collaboration and creativity dimensions. In the critical thinking dimension, the focus is on creativity and innovation, critical thinking and problem-solving, the ability to analyse complex problems, investigate unclarified matters and evaluate different perspectives or sources of in-formation, and arrive at appropriate conclusions based on evidence and reason (Ravitz et al., 2012; Toharudin, 2017; Tuzlukova and Prabhukanth, 2018). In the communication dimension, the focus is on listening skills as well as being able to communicate effectively using various oral, written, and digital tools in the communication dimension (Fullan, 2013). In the collaboration dimension, the focus is on working respectfully and effectively as a team to generate, use and share knowledge and innovating by providing solutions (Trilling and Fadel, 2012). Lastly, in the creativity dimension, the focus is on creative thinking skills in the context of the production of knowledge, including different ideas for social progress. Creativity is emphasized in all classifications of twenty-first-century skills. The third category is “Information, Media and Technology Skills,” which focuses on information literacy, media literacy, and information and communication technology (ICT) skills.

Language proficiency has been closely linked to communication in the modern world, which is the most fundamental building block for learning new knowledge and bringing about change. Accessing information and making use of the obtained information by analyzing it accurately, comprehension and expression skills, and language use are among the most basic components of twenty-first-century skills. The twenty-first-century skills also include reading, writing, interpretation, and synthesis skills (Ananiadou and Claro, 2009; Trilling and Fadel, 2012; Geisinger, 2016).

One of the core aspects of twenty-first-century skills is “language.” Language skills are important for all dimensions of twenty-first-century skills. The importance of language skills in the context of twenty-first-century skills was highlighted by the Modern Language Association (MLA). The re-port published by the MLA in 2007 suggested combining language teaching programmes with twenty-first-century skills. The MLA has highlighted the need to prepare a curriculum that will enable the students learning a second language to effectively communicate with native speakers through the effective use of the second language in question. In addition, MLA envisaged the development of a perspective that would enable students to under-stand the world in terms of another language (Geisler et al., 2007). The studies that ad-dressed the current situation in light of the MLA's report 10 years later (Lomicka and Lord, 2018; Cox and Montgomery, 2019) concluded that the curricular changes needed to support the development of twenty-first-century skills were not sufficiently implemented in most of the currently available language programmes, and a significant large-scale reform has yet to be achieved.

Twenty-first-century skills enable the individual in learning what is needed to be competent and qualified in the most efficient way (Louis, 2012; Hamarat, 2019). Learning twenty-first-century skills is not limited to educational environments. As a matter of fact, twenty-first-century skills can be more effectively acquired within the scope of lifelong learning. Individuals with twenty-first-century skills are expected to be productive, efficient, responsible, entrepreneurial, and social individuals with leadership qualifications who can think, communicate, analyze, and synthesize critically and creatively (Kurudayioglu and Taşkin, 2019).

One of the studies investigating the relationship between multiple intelligence types and twenty-first-century skills is by Ipekşen (2019). They found that multiple intelligence types predicted twenty-first-century skills. In addition to this result, it has been revealed that twenty-first-century skills of students can be developed with activities based on multiple intelligences. In studies investigating the relationship between multiple intelligences and twenty-first-century skills, emotional intelligence is addressed in particular. For example, many studies examining the effect of emotional intelligence on problem-solving skills argue that intelligence and problem-solving skills are related (Kim and Han, 2015; Aslan, 2019; Ndawo, 2021). Other intelligence types are also effective in the problem-solving skills of individuals. Intelligence types may change according to social, environmental and economic conditions, and this may affect people's problem-solving skills (Çinkiliç and Soyer, 2013). It is known that intelligence also has a positive effect on cooperation and leadership skills (Zhang et al., 2018). Similarly, in many studies investigating the relationship between creativity and intelligence, a highly significant relationship was found between intelligence types and creativity ability (Xu et al., 2019; Plucker et al., 2020; Frith et al., 2021). In another recent study (Uçar, 2021), the role of intelligence and creativity in the entrepreneurial tendencies of the Z generation was examined. According to the findings of the research, the creativity levels of the Z generation predict their entrepreneurial tendencies positively and significantly. In all the studies mentioned, the positive relationship between intelligence and twenty-first-century skills are emphasized.

To summarize, the concepts of twenty-first-century skills, intelligence types and learning technologies in the context of foreign language learning process were emphasized in this study. The primary goal of this study was to establish how well the concept of twenty-first-century abilities, as was discussed above, might predict the use of foreign language learning technology by higher education students. The second objective of this study was determined as to evaluate whether the perceived intelligence type differentiates higher education students' use of foreign language learning technologies.

### 2.3. Three variables of the research in the context of teaching Turkish as a foreign language

The International Society for Technology in Education (2017) Report drew attention to the relationship between intelligence types, twenty-first-century skills, and the use of technology for learning purposes. The ISTE 2017 report revealed that logical–mathematical intelligence is related to innovative skills, which are directly linked to technology. Innovation is one of the twenty-first-century skills. It is accepted that students who develop and improve their innovative skills can easily adapt to technology and construct knowledge. While adapting to technology, students can produce original ideas, analyse and evaluate their thoughts, and try different ways to solve the problems they encounter (Anagün et al., 2016). Innovative and applicable technological methods were proposed for use by teachers teaching Turkish as a foreign language with a view to making teaching Turkish more interesting, effective and enjoyable (Özkan et al., 2017). In another study, specific course activities were prepared in order to incorporate technology-based materials into teaching Turkish as a foreign language (Ural, 2016). It has been observed in all these studies that the use of low-cost technological materials increased the active participation of the students in the classes and in their willingness to learn. In other related study, Güler and Kalin Sali (2021) determined that the use of Edmodo positively affected university students' learning of Turkish as a foreign language. In short, as stated in studies by Liu and Xin (2018) and Zhao and Tianyuan (2019), it is self-evident that foreign language education must be supported with learning technologies. Mettursun (2018) addressed all types of intelligence in the context of teaching Turkish to foreigners and determined that taking multiple intelligence theory into account in teaching the language to foreigners positively affected students' Turkish learning and facilitated their acquisition of Turkish skills. In parallel, Tilbe (2006) determined that the students who learned Turkish from course materials prepared based on the multiple intelligence theory (the experimental group) were more successful than other students (the control group). Similarly, in a study where the effects of teaching practices based on multiple intelligence theory on Turkish reading comprehension skills were investigated, Epçaçan (2013) found that teaching Turkish language using different applications based on intelligence types was very effective in improving students' reading comprehension skills.

Eubanks et al. (2018) investigated whether the technology-integrated twenty-first-century writing workshop was effective for students' writing skills and attitudes, and determined as a result that their writing barriers decreased as they used technology within the scope of the technology-integrated twenty-first-century writing workshop. Bican (2021) discussed the opportunities offered by digital learning environments for writing skills in the context of teaching Turkish to foreigners and found that digital environments have contributed to students' writing skills in and outside the classroom. This finding was attributed to the fact that students were able to utilize their writing skills and receive feedback in virtual learning environments. Digital learning environments were also stated to increase students' problem-solving, critical thinking and creativity skills (Yilmaz et al., 2020, 2022; Atasoy, 2021). In a study by Güngör (2021), “Teaching Turkish as a Foreign Language in the Context of Twenty-First-Century Skills,” learning and innovative skills in addition to communication, cooperation, creativity and critical thinking skills were analyzed, and it was determined that Turkish lessons do not reflect contemporary approaches. Yilmaz and Babacan (2015) investigated podcast applications aimed at improving listening skills in teaching Turkish as a foreign language and found that they enriched students' listening skills and the process of teaching Turkish as a foreign language. In addition, in a study conducted with a view to increasing the listening comprehension success of students learning Turkish as a foreign language and reducing their listening anxiety, Berk and Açik (2021) concluded that e-audience-based activities increased the success of listening comprehension.

## 3. Objective and research questions

When the studies examining the relationship between research variables were examined, it was noticed that some subjects were not investigated. It is seen that the studies examined the relationship between intelligence types and twenty-first-century skills focus on emotional intelligence. It is worth investigating the nature of the relationship between intelligence types (other than emotional intelligence) and twenty-first-century skills. It is emphasized that a person's usage of technology may have anything to do with their family, their education, or even themselves. The link between technology and individual competence is the main topic of this study. It aims to reveal which intelligence type most affects the use of technology in foreign language education.

Information, media, and technology skills are one aspect of twenty-first-century abilities. It is anticipated that students who are highly motivated and skilled in this subject would use technology extensively. This study focused on the learning and innovation skills category of twenty-first-century skills, whereas the other two categories, namely, life and career skills and information, media and technology skills, were deliberately excluded from the scope of the research. This is because, based on the results of a vast number of studies available in the literature (Ertmer and Ottenbreit-Leftwich, 2010; Young, 2012; Chang and Chen, 2015; Garba et al., 2015; Koh et al., 2015; Eubanks et al., 2018; LaForce, 2018), it is expected that the use of technology in foreign language learning, which is the dependent variable primarily investigated in this study, would be related to information, media, and technology skills. In this study, the focus is on learning and innovation skills, which is another twenty-first-century skill area. Does having learning and innovation skills affect the use of technology in foreign language education? This study sought to answer that question. Twenty-first-century skills in the context of the education, learning and teaching process do not only imply technology competence or technology use. The original aspect of this study that distinguishes it from many other relevant studies available in the literature is that it focuses on learning and innovation skills rather than information, media and technology skills, which were already addressed numerous times in the context of technology use for foreign language learning. The research questions prepared based on this objective are as follows.

i. Is there a difference between self-perceived dominant intelligence types in terms of using learning technologies in Turkish language learning?

ii. Is there a difference between self-perceived dominant intelligence types in terms of students' twenty-first-century skills scores?

iii. Is there a correlation between twenty-first-century skills scale scores and Turkish language learning technologies scale scores?

## 4. Material and methods

In line with quantitative research design, this study utilized a descriptive approach. The basic feature of descriptive research is to study the current situation, in its own conditions and as it is. In this type of research, researchers are observers; they do not interfere or make any changes. The selection of the sample, the quality of the data collection tools and the accuracy of the data analysis are especially important in quantitative descriptive research (Bacon-Shone, 2013). This study was created using the correlational research model in accordance with the quantitative research technique. According to Creswell (2002), correlational design can be used to predict and explain the relationship between variables. Two or more variables are related and how they affect each other is found out in correlational design. The dependent variables of the research were as follows: “Foreign Language Learning Technologies Scale score” and “Twenty-First-Century Skills Scale score.” The relationships, if any, between the total Twenty-First-Century Skills Scale scores, scores obtained from the subscales of Twenty-First-Century Skills Scale and the Foreign Language Learning Technologies Scale scores were investigated by correlation and regression analysis. On the other hand, the independent variable of the research was “perceived intelligence type.” Accordingly, it was investigated whether the foreign language learning technologies scale scores and twenty-first-century skills scale scores of university students, one of the dependent variables, differed according to the perceived intelligence type.

### 4.1. Population and sample

Purposeful and convenience sampling methods were used to create the study sample. Accordingly, the university with the highest number of international students in Northern Cyprus, where the Turkish language is the medium of instruction and one of the authors of this study has been working, was selected. In this way, the research data could be easily accessed and the data collection phase could be completed in a fast and economical manner. The study sample consisted of 431 university students who took Turkish as a foreign language in the 2021–2022 academic year at the selected university.

The sociodemographic data of the students included in the study sample are shown in Table 1. 66.8 percent of the students participating in this research are female and 33.2 percent are male. The majority of the students participating in the research are between the ages of 18–21 (54.8%). Most of the students are from the Middle East (32.3%) and African (64.7%) countries. The departments where the students study are dentistry (13.7%), medicine (16.2%), nursing and health sciences (55.7%), and physiotherapy and nutrition/dietetics (14,4%).

**Table 1:** Sociodemographic characteristics of the university students who participated in the study.

	**Number (*n*)**	**Percentage (%)**
**Gender**		
Female	288	66.8
Male	143	33.2
**Age**		
18–21	236	54.8
22–25	140	32.5
26 and older	55	12.8
**Turkish proficiency levels** ^*^		
A1.1	143	33.2
A1.2	192	44.5
A2.1	40	9.3
A2.2	49	11.4
B1.1	7	1.6
**Which of the following grade ranges does the final grade you received from the**		
**Turkish course fall into?**		
0–50	129	29.9
51–60	72	16.7
61–70	86	20.0
71–80	49	11.4
81–90	53	12.3
91–100	42	9.7
**The geographical region of origin**		
Middle East	139	32.3
Africa	279	64.7
Other	13	3.0
**Have you ever resided in a Turkish-speaking country before?**		
Yes	108	25.1
No	323	74.9
**Department**		
Dentistry	59	13.7
Medicine	70	16.2
Nursing/health science/first and emergency aid	240	55.7
Nutrition and dietetics/physiotherapy & rehabilitation	62	14.4

* The Turkish proficiency levels have been determined in accordance with the Common European Framework of Reference for Languages (CEFR).

As can be seen in Table 1, the university students have been studying at different faculties and have different Turkish proficiency levels. In this way, maximum diversity could be achieved in the study sample. The Turkish proficiency levels shown in Table 1 have been determined in accordance with the Common European Framework of Reference for Languages (CEFR). Accordingly, students were divided into A1, A2, and B1 levels based on officially announced final grades and the results of the Turkish language proficiency exam carried out in the university where this study was conducted. Each language level consisted of two stages. Students who are successful in the exams pass to the next level and continue to learn the language by increasing their level.

### 4.2. Data collection tools

The scales of twenty-first-century skills and the foreign language-learning technologies were used to collect the research data. In addition to these scales, a personal information form was used to define the sample and perform the related statistical analyses.

Personal Information Form: This included questions about students' gender, age, nationality, Turkish proficiency level, the faculty and department in which they were enrolled, whether they lived in a Turkish-speaking country before, the final grade they received for the Turkish course, how often they used computers while learning Turkish and their weekly Internet usage time. The form consisted mostly of multiple-choice questions.

Self-Perceived Intelligence Types: In the personal information form, there is a question about the most important independent variable of the study, which is “self-perceived intelligence type.” Saban (2010), one of the researchers who has been working on multiple intelligence theory, mentioned many techniques, i.e., observation, anecdote recording and student self-assessment, that can be used to determine intelligence types in addition to scales. Saban asked the students about their perceptions of their intelligence type and to provide information based on their self-awareness. A self-assessment question in which students evaluate their own intelligence types and find the most dominant intelligence types is included in the personal information form. The questionnaire is taken from Selçuk et al. (2004). In this questionnaire, university students read 32 statements about 8 intelligence types and assign 0, 1, 2, 3, or 4 points to each statement. A high score for the statements means that it is suitable for the respondent, and a low score indicates that it is not appropriate. Then, respondents write their scores in the table and find the total score for each intelligence type. If the score is equal, they read the statements again and score. In the end, respondents find a single intelligence type that is dominant for them. In this study, the self-perceived intelligence type is a categorical variable and will be used as an independent variable. The dominant intelligence type of the respondents will be deter-mined and the analysis will be carried out with it. Intelligence types other than the dominant intelligence type of the respondents will not be used in the analysis.

The Twenty-First-Century Skills Scale: For this, the Survey Questionnaire of the Implementation of 4Cs (Critical Thinking, Communication, Collaboration, Creativity), which was developed by Eker (2020), was used. The scale consists of 40 items. All items were constructed using a positive sentence structure. Answer choices in each item were prepared in accordance with a five-point Likert-type rating. Accordingly, the following answer choices were included in each item: always true of me, usually true of me, some-what true of me, usually not true of me and never true of me. The Turkish validity and reliability studies of the scale were also conducted by Eker. *Validity:* Given that this study focused on learning and that the studies on the relationship between learning and twenty-first-century skills available in the literature employed only the learning and innovation skills-4Cs category of the twenty-first-century skills, only the “Learning and Innovation Skills” category of the twenty-first-century skills scale was considered in this study with reference to Eker's abovementioned work. The twenty-first-century skills scale developed by Eker consists of communication and collaboration (Communicate Clearly, Collaborate with Others, Think Interdependently), creativity and innovation (Think Creatively, Work Creatively with Others, Apply Past Knowledge to New Situations), critical thinking and problem solving (Think Critically, Make Judgements and Decisions, Ask Questions, Solve Problems), and reflection and awareness (Metacognition-Thinking About Our Thinking, Reflect and Synthesize) sub-dimensions. *Reliability:* The Cronbach's Alpha values of the subscales were 0.907 for communication and collaboration, 0.932 for creativity and innovation, 0.898 for critical thinking and problem solving, 0.918 for the reflection and awareness subscales, and 0.970 for the overall twenty-first-century skills scale. In this study, twenty-first-century skills score is a continuous variable and is used as a dependent variable.

Foreign Language-Learning Technologies Scale: The foreign language-learning technologies scale developed by Hayta (2014) was used. All items were constructed using a affirmative sentence structure. Answer choices in each item were prepared in accordance with a five-point Likert-type rating. Accordingly, the following answer choices were included in each item: never, rarely, sometimes, often, and always. The Turkish validity and reliability studies of the scale were also conducted by Hayta. *Validity:* The scale, which has no sub-dimensions, was developed as a single-factor scale consisting of 41 items. The exploratory factor analysis of the scale was repeated for Turkish Cypriots. The variances explained by the factors were reviewed based on the results of the exploratory factor analysis applied by principal component analysis and varimax transformation, and it was found that the foreign language-learning technologies scale had a single-factor structure with an Eigenvalue >1. It was observed that the factor load of 41 items on the scale was 0.5 or higher, and thus no item was removed from the scale. It was determined that the scale's single dimension explained 45.60% of the total variance. *Reliability:* The Cronbach's Alpha value of the scale was calculated as 0.984. In this study, foreign language-learning technologies score is a continuous variable and will be used as a dependent variable.

### 4.3. Data collection process

First, the researchers who developed the twenty-first-century skills scale and the foreign language-learning technologies scale, which were intended to be used in this study, were contacted *via* e-mail, and their permission was obtained. The study protocol was submitted to the scientific ethics committee of the university where this study was conducted and the required ethics committee approval was granted. The nine instructors who teach Turkish to international students were informed of the ethics committee's acceptance of the project and provided with the pertinent details. The research questions were constructed into an online scale and each faculty member was asked to share this online scale with their students. The purpose of the study and the consent form were included in the first section of the online scale. Only the students who wanted to participate in the study voluntarily were expected to fill out the online scale. The names of the students were not included in the forms, and both the faculty members and students were informed that the research data would be kept confidential.

### 4.4. Data analysis

SPSS 24.0 (Statistical Package for Social Sciences for Windows, version 24.0, IBM Corp., Armonk, NY, U.S., 2016) software was used in the statistical analyses of the quantitative data collected. The Kolmogorov–Smirnov test, Quantile–Quantile Plots (QQ plots) and Skewness and Kurtosis coefficients were used to determine whether the research data conformed to the normal distribution, and it was determined that the scores obtained from the scales did not conform to the normal distribution. Accordingly, descriptive statistics pertaining to the scores obtained from the scales were expressed using arithmetic mean and standard deviation values and minimum and maximum values.

Since the data set did not show a normal distribution, Spearman's correlation and Kruskal–Wallis *H*-test, which are non-parametric tests, were used. Spearman's correlation analysis was used to determine the relationship between two dependent variables, foreign language-learning technologies scale scores and twenty-first-century skills scale scores. With the Kruskal–Wallis *H*-test, the researchers investigated whether there were significant differences between dominant intelligence types in terms of levels of foreign language-learning technologies. The Kruskal–Wallis *H*-test was run twice. Similarly, the Kruskal–Wallis *H*-Test was used to investigate whether there were significant differences between dominant intelligence types in terms of levels of twenty-first-century skills. In each analysis, a Kruskal–Wallis *H*-test was run with one independent and one dependent variable. In cases where there was a significant difference, pairwise comparisons were performed with the Mann–Whitney *U*-test.

## 5. Results

In this section, first, descriptive statistics of the variables searched in the study are presented. Then, the results of the statistical analyses conducted are given in accordance with the order of the research questions presented in the Introduction. As seen in Table 2, the number of university students who stated that they are more competent in interpersonal and naturalistic intelligence areas was the highest, whereas the number of university students who stated that they are more competent in intrapersonal and visual-spatial intelligence areas was the lowest. As seen in Table 3, university students obtained higher scores on the twenty-first-century skills scale. As for the scores obtained from the subscales of the twenty-first-century skills scale, it was observed that the scores obtained from the critical thinking and problem-solving subscale were the lowest. On the other hand, the analysis of the scores obtained from the foreign language-learning technologies scale indicated that the students used technology at a moderate level (x = 2.72/5). The first research question aimed to reveal whether the foreign language-learning technologies scale scores differed by self-perceived intelligence type. As can be seen in Table 4, university students' foreign language-learning technology scale scores did not differ significantly by perceived intelligence type.

**Table 2:** Self-perceived intelligence types of university students.

	**Number (*n*)**	**Percentage (%)**
**In which intelligence area do you consider yourself more competent?**		
Bodily-kinesthetic intelligence	28	6.5
Interpersonal intelligence	95	22.0
Intrapersonal intelligence	36	8.4
Linguistic-verbal intelligence	50	11.6
Logical-mathematical intelligence	61	14.2
Musical intelligence	65	15.1
Naturalistic intelligence	89	20.6
Visual-spatial intelligence	7	1.6

**Table 3:** The scores university students obtained from the twenty-first-century kills scale and the foreign language learning technologies scale.

**Twenty-first century skills scale and its subscales**	***n***		**s**	**Min**	**Max**
Communication and collaboration subscale	431	4.15	0.62	2.15	5.00
Creativity and innovation subscale	431	4.11	0.70	2.00	5.00
Critical thinking and problem solving subscale	431	4.00	0.75	1.50	5.00
Reflection and awareness subscale	431	4.10	0.63	2.05	5.00
Total twenty-first-century skills scale	431	4.06	0.72	1.00	5.00
Foreign Language Learning Technologies Scale	431	2.72	0.86	1.00	5.00

**Table 4:** Kruskal Wallis *H*-test analysis of the scores university students obtained from the foreign language learning technology scale scores by the self perceived intelligence types.

	**Perceived intelligent types**	***N***	**Mean rank**		***df***	***P***
Foreign language learning technologies scale	Bodily-kinesthetic intelligence	28	216.57	12.534	7	0.084
	Interpersonal intelligence	95	247.28			
	Intrapersonal intelligence	36	229.93			
	Linguistic intelligence	50	228.84			
	Logical-mathematical intelligence	61	192.57			
	Musical intelligence	65	192.07			
	Naturalistic intelligence	89	203.48			
	Spatial intelligence	7	211.29			

The second research question examined whether scores on twenty-first-century skills varied according to the type of self-perceived intelligence. As can be seen in Table 5, university students' total twenty-first-century skills scale scores as well as the scores they obtained from the critical thinking and problem solving, and reflection and awareness subscales of the twenty-first-century skills scale differed significantly by the perceived intelligence types. Pairwise comparisons were made with Mann–Whitney *U*-test. The results of these comparisons are given in Tables 6–8. In order for the Mann–Whitney *U* tables not to be too long, only the results with significant differences are included. When Table 6 is examined, according to Mann–Whitney *U* analysis, critical thinking and problem-solving subscales scores of students with self-perceived musical intelligence type were significantly lower than students with all other intelligence types. Similarly, in the critical thinking and problem-solving subscale, the scores of students with self-perceived spatial intelligence type were significantly lower than those of students with many intelligence types (except naturalistic and musical). In Table 7, according to Mann–Whitney *U* analysis, reflection and awareness subscale scores of students with self-perceived musical intelligence type were significantly lower than students with most of the self-perceived intelligence types (except spatial, bodily and kinesthetic). Similarly, in the reflection and awareness subscale, the scores of students with self-perceived spatial intelligence type were significantly lower than students with many intelligence types (except naturalistic, musical, and bodily-kinesthetic). In Table 8, the Mann–Whitney *U*-test was performed on the basis of the twenty-first-century scale total scores and significant differences were observed in self-perceived musical and spatial intelligence scores. The twenty-first-century total scores of the students with the self-perceived musical intelligence type were significantly lower than the scores of the students with the other five intelligence types (interpersonal, intrapersonal, linguistic, logical mathematical, naturalistic). In the self-perceived spatial intelligence type, there was a significant difference in a single intelligence type. The total scores of twenty-first-century skills of the students with the self-perceived logical mathematical intelligence type were significantly higher than the students with the self-perceived visual intelligence type.

**Table 5:** Kruskal Wallis *H*-test analysis of the scores university students obtained from the twenty-first-century skills scale by the self perceived intelligence types.

	**Perceived intelligence type**	***N***	**Mean rank**	***df***	***X^2^***	***p***
Communication and collaboration subscale	Bodily-kinesthetic intelligence	28	202.63			
	Interpersonal intelligence	95	233.04			
	Intrapersonal intelligence	36	220.78			
	Linguistic intelligence	50	223.74			
	Logical-mathematical intelligence	61	233.87	7	11,366	0.123
	Musical intelligence	65	173.12			
	Naturalistic intelligence	89	216.41			
	Spatial intelligence	7	195.71			
Creativity and innovation subscale	Bodily-kinesthetic intelligence	28	202.07			
	Interpersonal intelligence	95	222.09			
	Intrapersonal intelligence	36	231.36			
	Linguistic Intelligence	50	225.04			
	Logical-mathematical intelligence	61	239.02	7	9,790	0.201
	Musical intelligence	65	181.02			
	Naturalistic intelligence	89	215.64			
	Spatial intelligence	7	174.21			
Critical thinking and problem solving subscale	Bodily-kinesthetic intelligence	28	218.38			
	Interpersonal intelligence	95	235.26			
	Intrapersonal intelligence	36	241.36			
	Linguistic intelligence	50	235.35			
	Logical-mathematical intelligence	61	227.19	7	18,318	0.011^*^
	Musical intelligence	65	166.07			
	Naturalistic intelligence	89	210.31			
	Spatial intelligence	7	115.00			
Reflection and awareness subscale	Bodily-kinesthetic intelligence	28	196.48			
	Interpersonal intelligence	95	231.36			
	Intrapersonal intelligence	36	231.19			
	Linguistic intelligence	50	222.29			
	Logical-mathematical intelligence	61	241.84	7	15,601	0.029^*^
	Musical intelligence	65	170.03			
	Naturalistic intelligence	89	219.33			
	Spatial intelligence	7	121.93			
Twenty-first century skills scale	Bodily-kinesthetic intelligence	28	203.52			
	Interpersonal intelligence	95	231.48			
	Intrapersonal intelligence	36	233.07			
	Linguistic intelligence	50	227.26			
	Logical-mathematical intelligence	61	238.57	7	18,124	0.011^*^
	Musical intelligence	65	169.08			
	Naturalistic intelligence	89	214.88			
	Spatial intelligence	7	141.00			

* p < 0.05.

**Table 6:** Pairwise comparisons of the critical thinking and problem solving subscale scores of university students obtained from the twenty-first-century skills scale by the self-perceived intelligence types.

**Domain binary**	***N***	**Mean rank**	**Sum of ranks**	***U***	***p***
Bodily kinesthetic	28	55.55	1,555.50	670.5	0.044^*^
Musical	65	43.32	2,815.50		
Bodily kinesthetic	28	19.82	555.0	47.000	0.034^*^
Spatial	7	10.71	75.0		
Interpersonal	95	90.56	8,603.5	2,131.5	0.001^*^
Musical	65	65.79	4,276.5		
Interpersonal	95	53.37	5,070.5	154.5	0.018^*^
Spatial	7	26.07	182.5		
Intrapersonal	36	62.25	2,241.0	765.0	0.004^*^
Musical	65	44.77	2,910.0		
Intrapersonal	36	24.04	865.50	52.500	0.015^*^
Spatial	7	11.50	80.50		
Linguistic	50	68.31	3,415.5	1,109.5	0.004^*^
Musical	65	50.07	3,254.5		
Linguistic	50	30.96	1,548.0	77.000	0.017^*^
Spatial	7	15.0	105.0		
Logical-mathematical	61	72.81	4,441.5	1,414.5	0.005^*^
Musical	65	54.76	3,559.5		
Logical-mathematical	61	36.45	2,223.5	94.500	0.016
Spatial	7	17.50	122.5		
Musical	65	68.05	4,423.0	2,278.0	0.024^*^
Naturalistic	89	84.40	7,512.0		

* p < 0.05.

**Table 7:** Pairwise comparisons of the reflection and awareness subscale scores of university students obtained from the twenty-first-century skills scale by the self perceived intelligence types.

**Domain binary**	***N***	**Mean rank**	**Sum of ranks**	***U***	***p***
Interpersonal	95	89.44	8,496.5	2,238.5	0.003^*^
Musical	65	67.44	4,383.5		
Interpersonal	95	53.18	5,052.5	172.5	0.033^*^
Spatial	7	28.64	200.5		
Intrapersonal	36	60.64	2,183.0	823.0	0.013^*^
Musical	65	45.66	2,968.0		
Intrapersonal	36	24.07	866.5	51.5	0.014^*^
Spatial	7	11.36	79.5		
Linguistic	50	66.24	3,312.0	1,213.0	0.019^*^
Musical	65	51.66	3,358.0		
Linguistic	50	30.75	1,537.5	87.500	0.032^*^
Spatial	7	16.5	115.5		
Logical-mathematical	61	74.15	4,523.0	1,333.0	0.001^*^
Musical	65	53.51	3,478.0		
Logical-mathematical	61	36.48	2,225.5	92.500	0.014^*^
Spatial	7	17.21	120.5		
Musical	65	67.62	4,395.0	2,250.0	0.018^*^
Naturalistic	89	84.72	7,540.0		

* p < 0.05.

**Table 8:** Pairwise comparisons of the twenty-first-century skills scale scores of university students obtained from the twenty-first-century skills scale by the self perceived intelligence types.

**Domain binary**	***N***	**Mean rank**	**Sum of ranks**	***U***	***p***
Interpersonal	95	89.66	8,518.0	2,217.0	0.002^*^
Musical	65	67.11	4,362.0		
Intrapersonal	36	60.72	2,186.0	820.0	0.013^*^
Musical	65	45.62	2,965.0		
Linguistic	50	66.76	3,338.0	1,187.0	0.013^*^
Musical	65	51.26	3,332.0		
Logical-mathematical	61	73.84	4,504.0	1,352.0	0.002^*^
Musical	65	53.80	3,497.0		
Logical-mathematical	61	36.36	2,218.0	100.0	0.022^*^
Spatial	7	18.29	128.0		
Musical	65	67.96	4,417.5	2,272.5	0.023^*^
Naturalistic	89	84.47	7,517.5		

* p < 0.05.

The third research question aimed to reveal the correlations between the scores obtained from the total twenty-first-century skills scale, from the subscales of the twenty-first-century skills scale and from the foreign language-learning technologies scale. As seen in Table 9, there was a weak yet statistically significant correlation between the total twenty-first-century skills scale scores and the foreign language-learning technologies scale scores in the positive direction. For a correlation coefficient to be interpreted, the *p* < 0.05. In this study, *r* < 0.2 was found and there was a very weak correlation (Akoglu, 2018). Accordingly, as students' twenty-first-century skills scale scores increased, their foreign language-learning technologies scale scores also increased. No statistically significant correlation was found between the scores obtained from the subscales of the twenty-first-century skills scale and the foreign language-learning technologies scale scores.

**Table 9:** Correlations between the total twenty-first-century skills scale scores, scores obtained from the subscales of the twenty-first-century skills scale, and the foreign language learning technologies scale scores.

**Foreign Language Learning Technologies Scale scores**			
**Twenty-first century skills**	***r***	***p***	***N***
Communication and collaboration subscale	0.072	0.137	431
Creativity and innovation subscale	0.088	0.069	431
Critical thinking and problem solving subscale	0.069	0.154	431
Reflection and awareness subscale	0.091	0.058	431
Total twenty-first-century skills scale	0.114^*^	0.018	431

* *p* < 0.05.

## 6. Discussion

This section is structured in two parts. The first section evaluates and discusses the issue of self-perceived intelligence kinds in teaching Turkish as a foreign language in light of the study's findings. In this section, both the subjects of the self-perceived intelligence type and twenty-first-century skills, and the self-perceived intelligence type and foreign language-learning technologies results are discussed in the context of teaching Turkish as a foreign language. The second part is introduced below.

### 6.1. Discussion related with self-perceived intelligence types

As can be seen in Table 2, the number of university students who stated that they are more competent in self-perceived interpersonal intelligence and naturalistic intelligence was the highest. This result is compatible with the finding that the highest mean score was obtained from the communication and collaboration subscale of the twenty-first-century skills scale (x = 4.15) (see Table 3). The finding that university students thought they were more competent in self-perceived interpersonal intelligence, which implied that they are open to social learning, should be taken into account in the foreign language-learning process. Hence, activities involving group work should be incorporated into the foreign language education curriculum. Along these lines, Tekiner (2005) found that interpersonal intelligence was the most dominant intelligence type in university students learning a foreign language and concluded that interpersonal intelligence is directly related to group learning activities. Similarly, the university students who participated in this study stated that they are more competent in interpersonal intelligence followed by naturalistic intelligence (see Table 2). This is a remarkable finding since it demonstrates the importance of learning experiences outside the classroom in foreign language teaching. Recently, Mousa (2022) revealed the positive effects of out-of-class teaching activities on foreign language learning. Several studies reported the positive effects of extracurricular and social activities on student motivation and language learning in the context of teaching Turkish language to foreigners (Kinay, 2017; Saydam and Çangal, 2018). These findings indicate that learning activities that activate the interpersonal intelligence type positively affect learning Turkish as a foreign language.

In this study, the number of students whose verbal-linguistic intelligence is dominant is less than the students whose interpersonal intelligence, nature intelligence, musical intelligence and mathematical intelligence are dominant (See Table 2). According to the results of the research, the rate of students whose verbal-linguistic intelligence is dominant is ~12%. However, in the literature, it is emphasized that students with verbal-linguistic intelligence will be more successful in the studies related to the foreign language learning process and the multiple intelligence areas of the students. Özkan (2008) and Trilling and Fadel (2012) determined that language abilities and potential were best expressed by verbal intelligence. Moreover, verbal intelligence predicts the flexibility, communication and cooperation skills included in the “Life and Career Skills” category of twenty-first-century skills, which prompt individuals to come together and share ideas. In parallel, in a study where collaborative tasks that can be applied in the online environment in teaching Turkish as a foreign language were emphasized, Inan (2021) found that collaborative dialogues performed in the target language in order to prompt learners talk to each other, listen to each other and write together helped learners control and support each other's language learning. In this way, the targeted acquisitions in learning the Turkish language were achieved with activities that enable both verbal intelligence and collaborative skills.

The results of this study indicated that interpersonal intelligence significantly affected the problem-solving dimension of twenty-first-century skills (See Tables 5, 6). Similarly, Kiremitçi et al. (2014) found a statistically significant positive relationship between university students' multiple intelligence areas and problem-solving skills. They determined that students with interpersonal intelligence in addition to logical and mathematical intelligence had better problem-solving skills. On the other hand, Kiremitçi et al. (2014) found that people with logical–mathematical intelligence in addition to verbal–linguistic, bodily–kinesthetic and naturalistic intelligence were more successful in solving problems. Students with high perceived levels of interpersonal intelligence were found to also have high critical thinking skills in this study (See Tables 5, 6). In parallel, Sardogan et al. (2006) found that students with high problem-solving skills also have high personal and social adaptation skills. Similarly, Dündar (2009) found a positive relationship between personal adjustment and problem-solving skills. In addition, in a study that investigated the relationship between teachers' multiple intelligence domains and their problem-solving skills, Genç (2012) found a positive correlation between teachers' intrapersonal intelligence and their problem-solving skills.

In this study, the twenty-first-century skill scores of the students in the two intelligence types related to art (musical and spatial) were lower than the students in the other intelligence areas (see Tables 6–8). Based on this result, it is necessary to examine the extent to which the definitions of twenty-first-century skills overlap with artistic development or artistic competencies. Although Erdoğan (2020) states that creativity, which is an important dimension in twenty-first-century skills, is also related to art, she emphasizes that the relationship between twenty-first-century skills and artistic skills needs to be examined in detail.

According to the findings in Table 4, it was revealed that having different intelligence areas did not differentiate the use of technology in learning Turkish as a foreign language. A similar result in this study, that there is no significant difference in the use of learning technologies according to multiple intelligence types, is in line with the findings of Balakrishnan and Gan's (2016) study. Balakrishnan and Gan (2016) investigated the effectiveness of students' learning styles, i.e., intelligence types, on technology use, and found that there are many different factors affecting it. In line with the results of Balakrishan and Lay's study, it was found in this study that the foreign language-learning technologies scale scores of the students did not differ significantly by perceived intelligence type. Studies addressing the theory of multiple intelligences in combination with learning technologies have generally focused on how the theory of multiple intelligences can be integrated into technology-oriented teaching. In these studies, it is emphasized that each student can be more successful in the learning process if enriched and various learning technologies that address all intelligence types are used (Gardner and Veenema, 1996). In parallel, Sahin Timar (2010) determined that the materials and web-based environments prepared in accordance with the theory of multiple intelligences thus addressed the dominant intelligence types of the students and increased students' success, by assisting in students' understanding of the subject, increasing students' interest in lessons, prompting students' active participation in classes and facilitating learning.

### 6.2. Discussion related with twenty-first-century skills and language learning technologies

In the second part of this discussion, the relationship between two dependent variables, except for the self-perceived intelligence type variable, is discussed. The relationship between twenty-first-century skills and language learning technologies in the context of teaching Turkish as a foreign language is examined on the basis of research findings. As can be seen in Table 2 in the Results section, university students scored an average of 4 points in the items of all the subscales of the five point Likert type twenty-first-century skills scale, indicating that they possess the necessary twenty-first-century skills. In parallel, Engin and Korucuk (2021) determined that the twenty-first-century skills of university students were high. Similarly, in several other studies university students were found to possess high twenty-first-century skills (Erdogan, 2018; Kozikoglu and Altunova, 2018). All kinds of learning activities that university students participate in throughout their lives in order to develop their knowledge, skills, interests and competencies, i.e., lifelong learning skills, can form the basis of twenty-first-century skills. The fact that university students were found to possess high twenty-first-century skills was attributed to lifelong learning (Erdogan, 2018; Kozikoglu and Altunova, 2018). In another study, it was emphasized that lifelong learning skills not only positively affect twenty-first-century skills but also increase academic success (Demirel, 2009). In contrast, some studies suggested that academic skills are slightly related or not related at all to twenty-first-century skills. In one of these studies, Göktepe Yildiz (2020) found that students' academic achievement levels were weakly correlated with some twenty-first-century skills including entrepreneurship-innovation, information technology literacy and career awareness, but not with other twenty-first-century skills including critical thinking, problem-solving, social responsibility and leadership. Similarly, as shown in Table 1, almost half of the university students who were learning Turkish as a foreign language stated that the final grade they received from the Turkish course was less than the passing grade (60 out of 100), which indicated that their academic success was low even though they were found to possess high twenty-first-century skills. The discrepancies between the findings of these studies can be attributed to the fact that foreign language learning requires lifelong learning skills, establishing connections between the content taught and daily life, and having learning experiences outside the classroom.

The results of this study revealed that university students have been using technology in foreign language learning at a moderate level (see Table 3, x = 2.72). The fact that the mean foreign language-learning technology scale score was found to be at a medium level despite the mean twenty-first-century skills scale score being high was attributed to students' use of technology for entertainment and killing time and not using it for learning. In parallel, Coşkun et al. (2007) found that even university students studying in academic programmes that require higher academic skills, such as medical education, use technology primarily for entertainment (42%), secondarily to communicate with each other (38%), and only tertiarily for learning, working on projects and homework (30%). The reasons underlying students' lower use of technology for learning are worth investigating, since understanding these reasons may guide educators and policymakers. Özdal et al. (2022) found that students who set learning goals, have the motivation and make the effort to develop learning strategies, and seek help to eliminate all kinds of problems they face during the process were more successful than other students in the online learning process. Thus, they concluded that the development of online self-regulation skills for students is as important as teachers' guidance in the use of technology for learning purposes.

The results of the correlation analyses given in Table 3 revealed that although the learning and innovation skills scores of the students were high, these scores did not relate to technology use for foreign language learning. More specifically, the 4Cs, namely, critical thinking, collaboration, communication and creativity skills, do not significantly affect the use of technology in the foreign language-learning process. This finding demonstrated the necessity of involving social skills in the development of technological skills. The effectiveness of technology-supported learning environments that involve social skills has been brought to the forefront in some studies (Nevgi et al., 2006; Günindi, 2014).

### 6.3. Limitations of the study

One of the study's limitations was that foreign students' Turkish language proficiency was assessed as a whole. Hence, further studies that address the effects of twenty-first-century skills, perceived intelligence types and learning technologies separately for each language skill, i.e., listening, speaking, reading and writing, in the context of teaching and learning Turkish as a foreign language would be useful. Secondly, the fact that only one dimension (learning and innovation skills-4C) of twenty-first-century skills was addressed in this study may be seen as another limitation. Therefore, further studies may address the other two dimensions (life and career skills and information, media and technology skills). Thirdly, the fact that the dominant intelligence types of the university students who participated in this study were determined based on students' own experiences and perceptions may be considered another limitation. The intelligence types of the university students who participated in this study were identified based on their perceptions (or self-assessment) did not allow detailed analysis of the results. The use of technology in language learning can be addressed in detail with a valid and reliable scale that assesses different intelligence types. In this way, it may be possible to further evaluate the relationship between intelligence types and the use of technology in foreign language learning.

## 7. Conclusions and recommendations

In conclusion, the results of the study revealed a weak yet statistically significant correlation between twenty-first-century skills and foreign language-learning technologies usage. Future studies may focus on the relationships between the other categories of twenty-first-century skills, namely, life and career skills and information, media and technology skills, and usage of foreign language-learning technologies. Additionally, students' scores in twenty-first-century skills differed significantly, whereas their scores for foreign language-learning technology did not, according to their perceived intelligence types. Based on the finding that the type of perceived intelligence makes a difference in twenty-first-century skills but not in language-learning technologies, it is important to in-crease the number of studies on the effectiveness of intelligence in learning a foreign language.

The other twenty-first-century skills that are thought to be related to intelligence in the literature should be researched in light of the fact that twenty-first-century skills and language-learning technologies have a poor relationship based on these research findings. It is important to develop twenty-first-century skills by associating them with all learning processes both in the school environment and outside the school environment, rather than thinking of them as skills to be taught. It can be inferred that the theory of many intelligences is related to talents that are valued today based on the conclusion that different types of intelligence make a difference in twenty-first-century skills. Thus, the theory of multiple intelligences is still up to date. In this respect, it can be said that teaching methods in multiple intelligence theory, textbooks and measurement–evaluation approaches can be developed and used in teaching Turkish as a foreign language. In this study, it was found that perceived intelligence type does not affect the use of learning technologies in learning Turkish as a foreign language. Thus, it can be suggested that research should be conducted based on other variables. For example, whether the environment in which the person lives or their willingness to learn affects the use of foreign language-learning technology can be investigated.

The pedagogical implications of this study can be summarized as follows. The research's findings indicate that students in higher education possess twenty-first-century skills. Based on this finding, it is possible to engage students in the courses and accomplish effective foreign language acquisition if foreign language education is carried out in accordance with modern methodologies and based on twenty-first-century abilities. According to the research findings, the students' use of technology in foreign language education is at a moderate level. Foreign language education courses could be planned for how students can use technology more effectively and more frequently while learning a language. Considering that there is a difference between using technology in daily life and using it for educational purposes, both scientific and applied studies should be carried out especially on technology-supported language education. In the study, it was observed that the type of interpersonal intelligence was high. It has been revealed in this study that it is important to include social learning rather than individual and competitive learning in foreign language education classes. Based on the high level of natural intelligence of the students, the necessity of conducting foreign language education lessons outside the classroom has emerged.

## Data availability statement

The raw data supporting the conclusions of this article will be made available by the authors, without undue reservation.

## Ethics statement

The study was conducted in accordance with the Declaration of Helsinki, and approved by the Institutional Review Board (or Ethics Committee) of Cyprus International University (EKK21-22/011/0010 and 18.03.2022) for studies involving humans. The patients/participants provided their written informed consent to participate in this study.

## Author contributions

EKK: conceptualization, resources, visualization, methodology, formal analysis, and data curation. EKK and AG: investigation, writing—original draft preparation, and writing—review and editing. All authors contributed to the article and approved the submitted version.
